# Functionalized γ-Boehmite Covalent Grafting Modified Polyethylene for Lithium-Ion Battery Separator

**DOI:** 10.3390/ma17092162

**Published:** 2024-05-06

**Authors:** Yuanxin Man, Hui Nan, Jianzhe Ma, Zhike Li, Jingyuan Zhou, Xianlan Wang, Heqi Li, Caihong Xue, Yongchun Yang

**Affiliations:** 1 Qinghai Provincial Key Laboratory of New Light Alloys, School of Mechanical Engineering, Qinghai University, Xining 810016, China; 13841637639@163.com (Y.M.); nanhui@qhu.edu (H.N.); 15613418871@163.com (J.M.); 17638373625@163.com (Z.L.); 2 Qinghai Beijie New Material Technology Co., Ltd., Xining 810016, China; zhoujingyuan@pulead.com.cn (J.Z.); wangxianlan@pulead.com.cn (X.W.); 3 School of Materials Science and Engineering, Harbin Institute of Technology, Harbin 150080, China; liheqi2023@126.com; 4 Qinghai Institute of Science and Technology Information Co., Ltd., Xining 810016, China

**Keywords:** γ-boehmite, covalent grafting, interfacial engineering, high electrolyte affinity

## Abstract

In the field of lithium-ion batteries, the challenges posed by the low melting point and inadequate wettability of conventional polyolefin separators have increased the focus on ceramic-coated separators. This study introduces a highly efficient and stable boehmite/polydopamine/polyethylene (AlOOH-PDA-PE) separator. It is crafted by covalently attaching functionalized nanosized boehmite (γ-AlOOH) whiskers onto polyethylene (PE) surfaces. The presence of a covalent bond increases the stability at the interface, while amino groups on the surface of the separator enhance the infiltration of the electrolyte and facilitate the diffusion of lithium ions. The PE-PDA-AlOOH separator, when used in lithium-ion batteries, achieves a discharge capacity of 126 mAh g^−1^ at 5 C and retains 97.1% capacity after 400 cycles, indicating superior cycling stability due to its covalently bonded ceramic surface. Thus, covalent interface modification is a promising strategy to prevent delamination of ceramic coatings in separators.

## 1. Introduction

Lithium-ion batteries are highly promising energy storage devices, celebrated for their compact size, high voltage, substantial energy density, low self-discharge rate, lack of memory effect, impressive specific energy, broad operating temperature range, and ability to charge and discharge quickly at high rates. Additionally, they cause minimal environmental pollution, making them ideal for integrating renewable resources with high-power applications [1,2,3,4]. The separator plays a crucial role in lithium-ion batteries, functioning to prevent the positive and negative electrodes from making direct contact, thereby averting short circuits. Additionally, the separator enables the free movement of lithium ions within the electrolyte between the positive and negative electrodes, thus supporting the electrochemical process of charging and discharging [5]. The thickness of the separator typically ranges from 10 to 25 μm. Thinner separators can enhance the energy density of the battery, but excessively thin separators may compromise mechanical strength and puncture resistance. A porosity above 40% aids in improving ion transmission efficiency, thereby increasing the battery’s conductivity and enhancing charge–discharge performance. The general pore size should be between 0.01 and 0.1 μm. Separators require good wettability to ensure that the electrolyte can fully penetrate and cover the entire surface of the separator. Separators must possess sufficient mechanical strength (tensile strength > 98 MPa) and thermal stability to withstand the physical and thermal stresses during manufacturing and long-term operation. Additionally, separators should have good chemical stability to remain stable in the electrolyte environment [6]. At present, the separators that are most commonly employed are porous polyethylene (PE) and polypropylene (PP) [7,8]. While polyolefins are effective materials for separators, they also exhibit significant drawbacks. They are difficult to wet by the electrolyte, which hinders battery performance. Additionally, PE and PP have relatively low melting points, with significant shrinkage occurring between 130 and 160 °C. In the event of abnormal battery heating, this can easily lead to safety issues [9,10,11,12]. To address the aforementioned issues, applying a layer of inorganic particles to the surface of polyolefin separators (such as MOF [13], SiO_2_ [14,15,16,17,18,19], Al_2_O_3_ [20,21,22], SiO_2_/Al_2_O_3_ particle mixtures [23], TiO_2_ [24,25], metal hydroxides [26], γ-AlOOH [27,28,29,30], etc.) is an economical and easily achievable method. Powders that possess surface hydrophilicity improve the compatibility of composite separators with the electrolyte, thereby enhancing the electrochemical performance of the battery. Moreover, inorganic particles need to exhibit robust thermal stability and adequate mechanical strength to ensure battery safety. Among these developments, separators coated with alumina have already been introduced to the market. YM Deng prepared Al_2_O_3_-PVDF-HFP-CMC/PE (Al-PHC/PE) composite separators using a hot-pressing method [31]. The employment of polyvinylidene fluoride–hexafluoropropylene (PVDF-HFP) and carboxymethyl cellulose (CMC) as binders has facilitated the creation of an Al_2_O_3_ coating. Using PVDF-HFP as a binder significantly improves the mechanical strength and ionic conductivity of the composite separator, thanks to the aggregation of Al_2_O_3_ nanoparticles. Although the use of binders can somewhat improve the wettability and thermal stability of lithium-ion batteries, they also increase the thickness of the separator, which might negatively affect the battery’s electrochemical performance. Additionally, surfaces coated with inorganic ceramic particles may delaminate from the separator, leading to decreased separator performance and pore blockage, which impedes lithium-ion transport. The ceramic coating also extends the ion transport distance, occupies more battery space, and reduces the volume of active materials, which contradicts the high energy density sought in lithium-ion batteries. Therefore, developing advanced ceramic composite membrane preparation techniques with more stable and efficient electrochemical performance is crucial for enhancing the safety and rapid charge–discharge capabilities of lithium-ion batteries [32,33]. 

Due to their robust adhesive strength and superior stability, covalent bonds are extensively used across various domains, such as surface modifications, energy storage, and catalysis, thanks to their dependable binding forces [34,35,36]. X Song [37] used chemical self-assembly to form a thin MnO_2_ layer on a conventional PE membrane. Because of the existence of oxygen-containing groups, covalent bonds were formed between the MnO_2_ nanoparticles and the PE separator, resulting in strong chemical adsorption. The dense structure and catalytic activity of the MnO_2_ layer improved the reversible capacity and adhesive strength. The battery constructed using the MnO_2_@PE separator demonstrated a cycle decay rate of 0.059% per cycle. Wang [38] employed an eco-friendly self-assembly technique using polyethyleneimine (PEI) and silica to create an ultrathin layer on commercial PE separators. The minimal increase in thickness of the composite separator relative to the original PE separator maintains the porous structure of the PE membrane. Following the addition of the PEI/SiO_2_ layer, the electrochemical performance was significantly improved, resulting in an elevated Li^+^ migration number. The enhanced PE separator demonstrated a strong capacity retention rate of 90.1% after 100 cycles at a 0.5 C rate. Additionally, the modification through covalent bonding grafting enhances the ion conductivity of the separator, thus improving the battery’s charging rate and cycle life.

γ-AlOOH, as a hydrate of aluminum hydroxide, holds great potential for a wide range of applications, particularly in battery technology [39]. Boehmite can effectively enhance the performance of battery separators, increasing their mechanical stability, as well as improving their thermal stability and resistance to lithium dendrites. While γ-AlOOH is commonly utilized as a coating to modify lithium-ion battery separators, there is limited documentation on covalent grafting methods.

The article introduces a covalent grafting interface engineering strategy. Initially, the original PE membrane surface is pre-treated with dopamine to generate abundant -OH groups. Subsequently, KH550 treatment is employed to generate rich amino groups on the surface. Simultaneously, KH560 is used to pre-treat the γ-AlOOH surface, introducing epoxy groups. Finally, through the ring-opening reaction between epoxy and amino groups, covalent bonds are formed between γ-AlOOH and the PE membrane, successfully creating the PE-PDA-AlOOH composite separator. This covalent grafting achieves increased interface stability of the composite separator without the involvement of adhesives. Moreover, the covalently bonded nano-γ-AlOOH layer does not significantly increase the thickness of the separator, preventing the separator from occupying more space within the battery and reducing the energy density of the battery.

## 2. Experimental Section

### 2.1. Materials

The following materials were used in the study: PE membrane (Qinghai North New Materials Technology Co., Ltd., Xining, China), Lithium hexafluorophosphate (1 M LiPF_6_ in EC:DEC:DMC = 1:1:1 *v*/*v*) solution, acetylene black, N-methyl-2-pyrrolidone (NMP AR, >99%; Shanghai Aladdin Biochemical Technology Co., Ltd., Shanghai, China), LiFePO_4_ (Suzhou Duodu Chemical Technology Co., Ltd., Suzhou, China), polyvinylidene fluoride (PVDF; Suwei Shanghai Co., Ltd., Shanghai, China), dopamine hydrochloride (C_8_H_12_ClNO_2_; AR, Shanghai Aladdin Biochemical Technology Co., Ltd., Shanghai, China), anhydrous ethanol (C_2_H_6_O; AR, Sinopharm Chemical Reagent Co., Ltd., Shanghai, China), deionized water (DI), 3-glycidoxypropyltrimethoxysilane (KH560; AR, Shanghai Aladdin Biochemical Technology Co., Ltd., Shanghai, China), 3-aminopropyltrimethoxysilane (KH550; AR, Shanghai Aladdin Biochemical Technology Co., Ltd., Shanghai, China), and lithium metal (Li, Tianjin CATL New Energy Materials Co., Ltd., Tianjin, China).

### 2.2. Preparation Methods

#### 2.2.1. Preparation and Functionalization of γ-AlOOH

γ-AlOOH was prepared using a hydrothermal method. AlCl_3_·6H_2_O was dissolved in deionized water and stirred for 2 h until complete hydrolysis. Then, 1 mol/L NaOH solution was gradually added into the above solution until a white precipitate formed, reaching a solution pH of 4. The mixed solution was transferred to a hydrothermal reactor and reacted at 200 °C for 24 h, followed by natural cooling to room temperature. After filtration, the precipitate was washed three times with deionized water, then dried at 80 °C for 12 h to remove residual moisture, and finally ground into powder. The γ-AlOOH powder was dispersed in anhydrous ethanol and KH560 was added dropwise (AlOOH/KH560 mass ratio of 1/2). Under nitrogen protection, the mixture was stirred at room temperature for 10 h. It was then washed with ethanol and dried.

#### 2.2.2. Preparation of PE-PDA-AlOOH and PE/AlOOH

**Figure 1:**
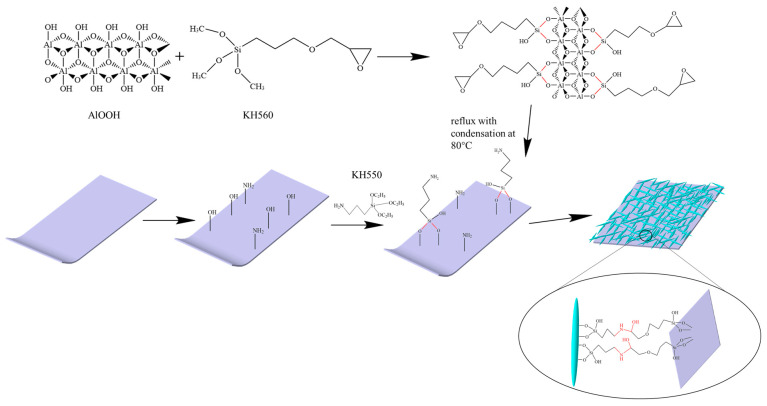
Experimental procedure for the PE-PDA-AlOOH separator.

The PE-PDA-AlOOH membrane was prepared through the ring-opening reaction between the new functional group amino on the surface of the PE membrane and the epoxy group on the functionalized γ-AlOOH surface, as shown in Figure 1. Firstly, the PE membrane was immersed in a 2 mg/mL dopamine solution (with methanol/buffer solution = 1/1 by vol as the solvent) for 24 h, followed by washing and air-drying. Then, the PE membrane with -OH groups was soaked in KH550 solution (KH550/anhydrous ethanol = 1/20 by vol) for 8 h and subsequently dried. Finally, the amino-functionalized PE membrane was immersed with the epoxy group-bearing γ-AlOOH in an ethanol solution at 80 °C for 8 h to obtain the PE membrane covalently modified by γ-AlOOH.

The preparation of PE/AlOOH began by dissolving 10 wt% γ-AlOOH and 1 wt% PVDF in NMP, stirring for 12 h. Subsequently, the mixed solution was uniformly coated on both sides of the PE membrane using a coater and dried for 24 h at 60 °C. The preparation process is shown in Figure 1. 

### 2.3. Preparation of Electrodes and Cells

Using a theoretical specific capacity of 172.5 mAh/g for LiFePO_4_ as the active material, PVDF as the binder, and adding a mixture of acetylene black and an appropriate amount of NMP, the components were mixed for twenty minutes (LiFePO_4_/PVDF/acetylene black = 8:1:1) to obtain a viscous solution. The solution was uniformly coated on aluminum foil and dried at 120 °C under vacuum for 12 h. The electrode sheets were cut into circular pieces with a diameter of 14 mm. Using metallic Li as the counter-electrode and 1 mol/L LiPF_6_ solution (EC:DEC:DMC = 1:1:1 *v*/*v*) as the electrolyte, LiFePO_4_/separator–electrolyte/Li, Li/separator–electrolyte/SS (stainless steel), and SS/separator–electrolyte/SS button cells were assembled in an argon-filled glove box (H_2_O and O_2_ contents both <1 ppm).

### 2.4. Characterization

The morphologies of the boehmite, separators, and their cross-sections were investigated using field emission scanning electron microscopy (FE-SEM, JEOL, Tokyo, Japan). The crystal structure of the products was analyzed through X-ray diffraction (XRD, PANalytical, Alemlo, The Netherlands). The bonding situation of the products was characterized using Fourier transform infrared spectroscopy (FT-IR, Rayleigh, Beijing, China) and X-ray photoelectron spectroscopy (XPS, Thermo Fisher Scientific, Shanghai, China). Samples measuring 50 mm in length and 20 mm in width were subjected to mechanical property testing using an intelligent electronic tensile machine. Thermal stability studies were conducted at different temperatures for 0.5 h in a vacuum drying oven, and the thermal shrinkage rate was calculated by comparing the area changes before and after heat treatment. A 10 μL water droplet was placed on the sample surface, and its contact angle was measured using a contact-angle goniometer after 5 s to determine its wetting performance.

#### 2.4.1. Porosity Testing

The separators were cut into equally sized circular pieces using a button cell cutter and then soaked in *n*-butanol solvent for 2 h to reach saturation. The weight change of the separator before and after immersion was recorded. The porosity was calculated using Equation (1) [40].

(1) $$Porosity\left(\%\right)=\frac{m_{1}-m_{0}}{\rho_{n}V}\times 100$$

where *m*_0_ and *m*_1_ represent the initial mass of the separator and the mass after soaking in *n*-butanol, *ρ_n_* is the density of *n*-butanol, and *V* is the volume of the separator.

#### 2.4.2. Liquid Absorption Testing

The separator was immersed in the electrolyte for 6 h and then excess electrolyte on the surface of the separator was wiped off with filter paper. The liquid absorption rate was calculated using Equation (2).

(2) $$Electrolyteuptake\left(\%\right)=\frac{m_{1}-m_{0}}{m_{0}}\times 10$$

where *m*_0_ and *m*_1_ represent the initial weight of the separator and the weight after soaking in the electrolyte, respectively.

#### 2.4.3. Ion Conductivity Testing

Using an electrochemical workstation at a constant voltage of 10 mV, the SS/separator–electrolyte/SS cell was scanned at a frequency of 0.01 to 10^6^ to obtain the separator cell resistance (Rb). The ion conductivity was calculated using Equation (3):

(3) $$\sigma =\frac{d}{R_{b}\times S}$$

where *S* represents the membrane area, *d* represents the membrane thickness, and *R_b_* is the membrane resistance.

The battery underwent LSV (linear sweep voltammetry) testing with a structure of SS/separator–electrolyte/Li. Following that, an electrochemical workstation was employed for stability testing with a voltage range of 2–7 V and a scanning rate of 0.5 mV/s.

#### 2.4.4. Cyclic Voltammetry

Cyclic voltammetry was performed on the LiFePO_4_/separator–electrolyte/Li cell to observe the electrochemical reactions of the electrode material during the lithium extraction and insertion process. The voltage range of the lithium battery was 2.5 V to 5 V, and the scan rate was 0.1 mV/s.

#### 2.4.5. Battery Performance Testing

The LiFePO_4/_separator–electrolyte/Li battery was tested using the LAND-CT2001C battery testing system manufactured by Wuhan Land Electronic Co., Ltd., Wuhan, China. The battery was tested at room temperature at 2 C, maintaining the voltage within the range of 2.5 V to 4.3 V, to assess charge–discharge stability. To further investigate the rate capability of the half-cell, charge–discharge tests were conducted at rates ranging from 0.2 C to 5 C.

## 3. Results and Discussion

**Figure 2:**
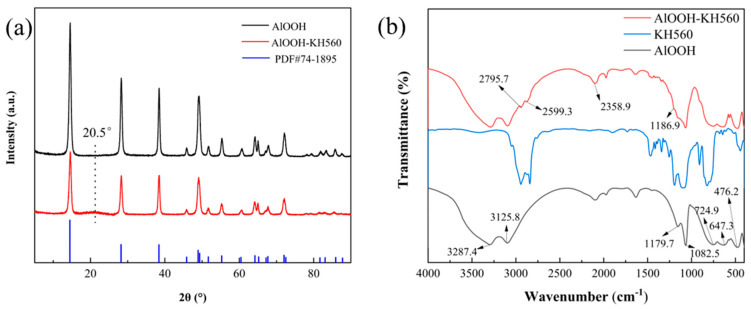
XRD (**a**) and FTIR (**b**) spectra of AlOOH and AlOOH-KH560.

The XRD patterns of the hydrothermally obtained γ-AlOOH and the functionalized γ-AlOOH are shown in Figure 2a. From the figure, it is evident that the diffraction patterns of the γ-AlOOH and AlOOH-KH560 samples are nearly identical, matching the standard spectrum of γ-AlOOH (PDF#74-1895), confirming the obtained product as γ-AlOOH [41]. AlOOH-KH560 exhibits a faint broad peak around 2θ = 20.5°, indicating the presence of an amorphous phase derived from the C in KH560 [42]. Figure 2b shows the FT-IR spectrum of the obtained product. In the figure, γ-AlOOH exhibits strong absorption peaks at 3287.4 cm^−1^ and 3125.8 cm^−1^, corresponding to the asymmetric and symmetric stretching vibration peaks of -OH. The strong absorption peaks at 1179.7 cm^−1^ and 1082.5 cm^−1^ correspond to the asymmetric and symmetric bending vibration peaks of -OH. Additionally, the peaks at 724.9 cm^−1^, 647.3 cm^−1^, and 476.2 cm^−1^ correspond to the twisting, stretching, and bending vibration peaks of Al-O, respectively [43]. In the infrared spectrum, the peaks at 2795.7 cm^−1^ and 2599.3 cm^−1^ in the AlOOH-KH560 sample correspond to the stretching vibrations of the C-H bonds in the CH_2_ groups of KH560 [44]. The peak at 1254.4 cm^−1^ is indicative of the stretching vibration of the C-O bond in the epoxy group. The peak at 1186.9 cm^−1^ corresponds to the stretching vibration of the C-C bond [45,46]. The 1000–1200 cm^−1^ region corresponds to the stretching vibration peak of Si-O-R [47], indicating a condensation reaction between the -OH of γ-AlOOH and the silane oxygen of KH560, demonstrating the successful grafting of KH560 onto γ-AlOOH. 

**Figure 3:**
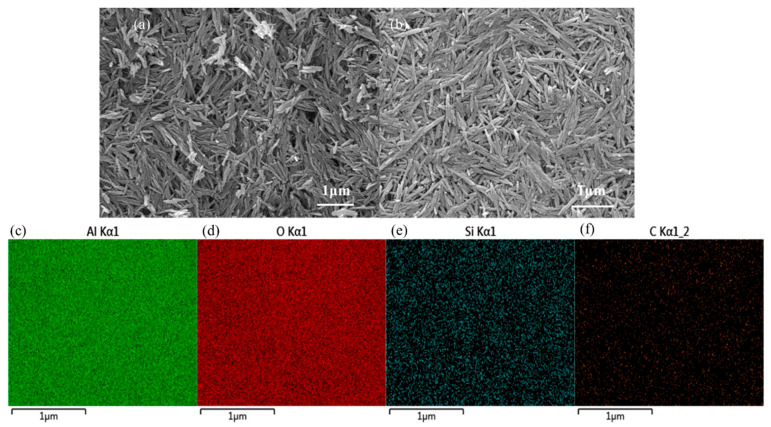
SEM images of γ-AlOOH (**a**) and AlOOH-KH560 (**b**) and EDS images of AlOOH-KH560 (**c**–**f**).

Figure 3a,b, respectively, show the SEM images of γ-AlOOH and AlOOH-KH560. It can be observed from the figures that the addition of KH560 does not significantly alter the morphology of γ-AlOOH. The EDS images of AlOOH-KH560 in Figure 3e,f reveal the uniform distribution of Si and C on γ-AlOOH, further confirming the successful synthesis of AlOOH-KH560. The elemental content in AlOOH-KH560 is presented in Table 1.

**Figure 4:**
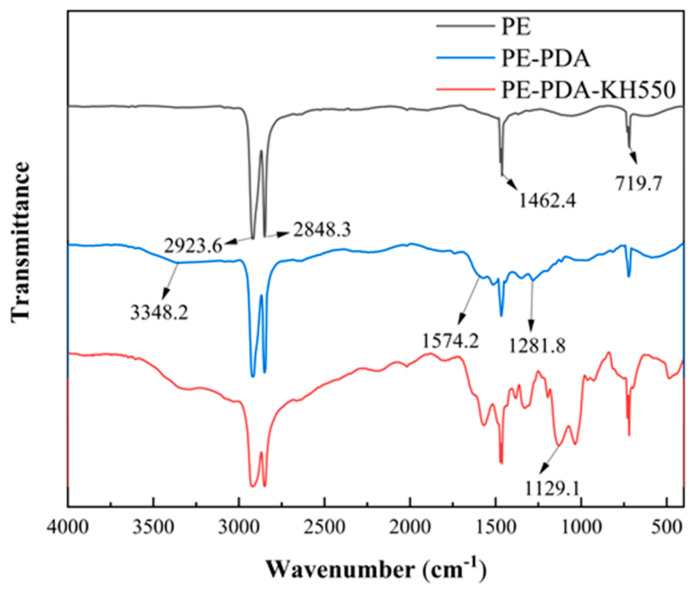
FT-IR spectra of PE, PE-PDA, and PE-PDA-KH550 membranes.

As depicted in Figure 4, The FT-IR analysis of the membrane surface composition revealed characteristic peaks for the PE base film: asymmetric stretching vibration of -CH_2_- at 2923.6 cm^−1^, symmetric stretching vibration of -CH_2_- at 2848.3 cm^−1^, bending vibration of -CH_2_- at 1462.4 cm^−1^, and bending vibration of -(CH_2_)_4_- at 719.7 cm^−1^. For the PE-PDA membrane, new peaks appeared: OH stretching vibration in PDA at 3348.2 cm^−1^, benzene ring stretching vibration at 1574.2 cm^−1^, and characteristic peaks due to N-H and C-N coupling vibration at 1281.8 cm^−1^, confirming PDA polymerization on the PE surface. In the PE-PDA-KH550, Si-OH stretching vibration at 3291.6 cm^−1^ overlaps with the PE-PDA’s -OH vibration peak, and the Si-O-Si characteristic absorption peak at 1129.1 cm^−1^ indicates successful grafting of KH550 onto the PDA-polymerized PE membrane.

**Figure 5:**
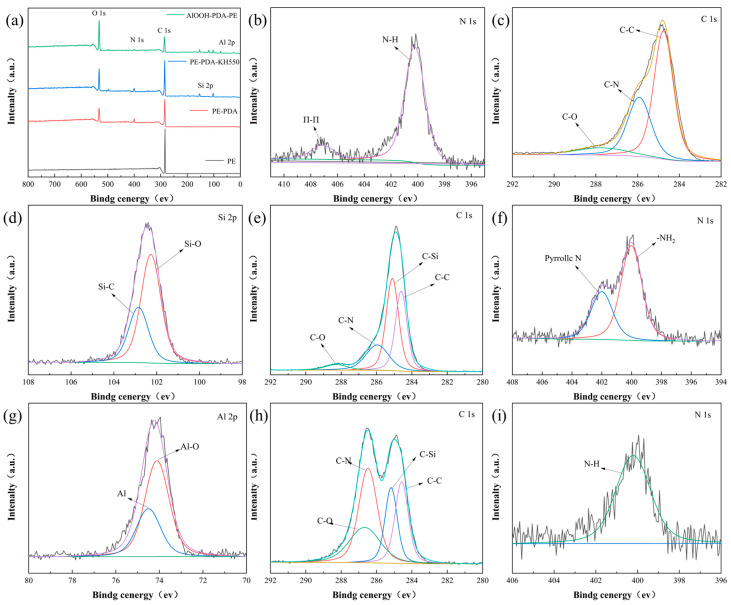
XPS full spectra of the membrane (**a**) before and after surface modification; N 1s (**b**) and C 1s (**c**) spectra of PE-PDA; Si 2p (**d**), C 1s (**e**), and N 1s (**f**) spectra of PE-PDA-KH550; Al 2p (**g**), C 1s (**h**), and N 1s (**i**) spectra of AlOOH-PDA-PE.

To identify the chemical composition information of the surface interface modification of the PE-PDA-AlOOH membrane, X-ray photoelectron spectroscopy (XPS) analysis was performed on the membrane. In Figure 5a, only the C element is present in the XPS of the PE separator. After the PDA treatment of the PE surface, N 1s and O 1s peaks were detected. The precise analysis of C 1s and N 1s in Figure 5b,c indicates peaks at 284.8 eV, 285.5 eV, and 400.3 eV, attributed to C-O, C-N, and pyrrolic N, respectively, suggesting the oxidative polymerization of PDA, leading to the formation of -OH on the PE surface. In comparison, the PE-PDA-KH550 membrane in Figure 5d–f shows the presence of -NH_2_ from KH550 at 400.3 eV in addition to the features of PE-PDA. Figure 5g shows the appearance of the Al-O binding peak at 74.3 eV in the AlOOH-PDA-PE membrane, and in Figure 5i, the N-H binding peak is located at 399.9 eV, indicating the successful covalent fixation of γ-AlOOH on the PE membrane surface.

**Figure 6:**
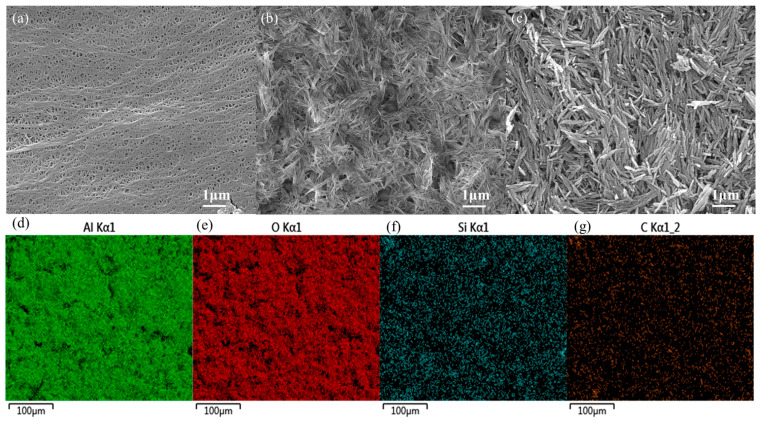
The SEM images of PE (**a**), PE/AlOOH (**b**), and PE-PDA-AlOOH (**c**), as well as the EDS mapping of PE-PDA-AlOOH (**d**–**g**).

Figure 6a–c are SEM images of the membrane. From Figure 6a, it is apparent that the surface of the PE membrane is covered with uneven, interconnected micro- and submicron pores. Figure 6b,c display similar surface structures for the PE/AlOOH and PE-PDA-AlOOH membranes, where γ-AlOOH is distributed on the surface of the PE membrane, forming a nanocrystalline whisker-like microstructure similar to that prepared by electrospinning. The γ-AlOOH whiskers create numerous pore structures on the PE surface, enabling the membrane to absorb and store more electrolyte, which is conducive to the conduction of lithium ions. EDS mapping of the membrane surface in Figure 6d indicates a uniform distribution of Al, O, Si, and C elements, further confirming successful grafting of γ-AlOOH onto the PE without the involvement of any adhesives. The elemental content in PE-PDA-AlOOH is presented in Table 2.

**Figure 7:**
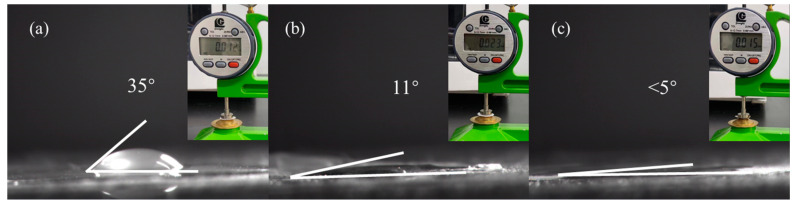
Contact angles and thickness of PE (**a**), PE/AlOOH (**b**), and PE-PDA-AlOOH (**c**) separators.

In separators for lithium-ion batteries, affinity and wettability with the electrolyte are crucial to ensuring fast ion transmission and low interfacial resistance in the battery. Contact-angle experiments were conducted on the PE, PE/AlOOH, and PE-PDA-AlOOH separators, as shown in Figure 7. Evidently, the contact angle of the PE-PDA-AlOOH separator (<5°) is much smaller than those of the PE separator (35°) and the PE/AlOOH separator (11°). This indicates that the PE-PDA-AlOOH separator has excellent electrolyte affinity and wettability. As shown in Table 3 and the inset in the upper right corner of Figure 7, the thickness of the PE-PDA-AlOOH separator is only 15 μm, which is not significantly thicker than the PE separator (12 μm) and significantly thinner than the PE/AlOOH separator (23 μm), reducing the volume of the separator. The porosity and electrolyte absorption rate of the PE-PDA-AlOOH separator are 67% and 164%, respectively, higher than those of the PE separator (41% and 107%) and the PE/AlOOH separator (57% and 150%). This is because the surface of the PE separator is non-polar, lacking polar groups, and has poor wetting with the electrolyte. The improved wettability of the modified separator with the electrolyte is due to the abundant polar -OH groups in Boehmite, which can enhance the interface compatibility between the separator and the electrolyte. Furthermore, the covalent bonds in the PE-PDA-AlOOH separator contain -NH and hydroxyl groups. These groups can intertwine with the molecular structure on the PE surface through hydrogen bonding, van der Waals forces, and other interaction forces, forming a more robust connection [48]. These functional groups can enhance the hydrophilicity of the separator. Improved hydrophilicity helps to increase the wettability of the electrolyte, thereby increasing the migration rate of lithium ions and the ionic conductivity of the battery.

**Figure 8:**
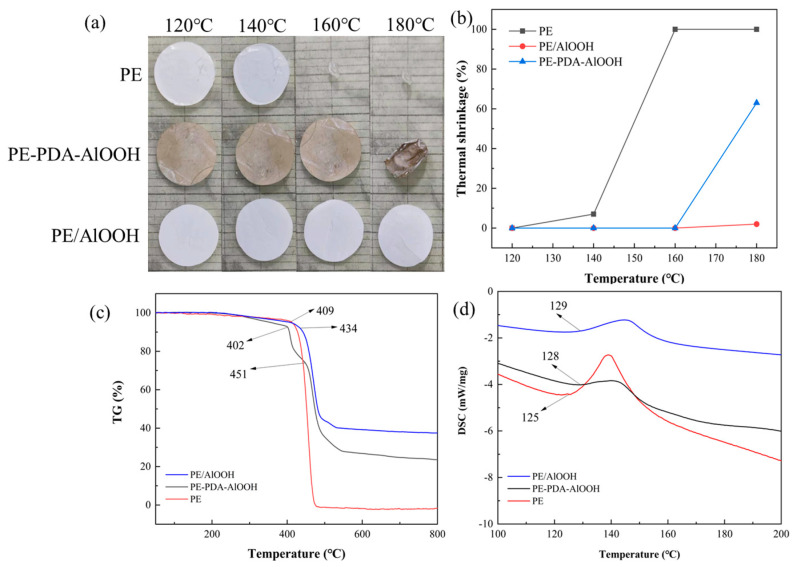
The stability of different separators under different temperature conditions for 0.5 h (**a**), thermal shrinkage rates (**b**), thermogravimetric (TG) curves (**c**), and differential scanning calorimetry (DSC) curves (**d**).

The thermal stability of the separator is closely related to the safety of the battery. The results of exposing separators to different temperatures for 0.5 h are shown in Figure 8a. It can be observed that the PE separator began to shrink at 140 °C and significantly melted at 160 °C. In contrast, no significant changes were observed for both the PE/AlOOH and PE-PDA-AlOOH separators, with the PE-PDA-AlOOH beginning to shrink significantly only at 180 °C. This indicates a substantial improvement in the thermal stability of the PE-PDA-AlOOH separator compared to the PE separator, as shown by the thermal shrinkage rate in Figure 8b. The TG and DSC curves in Figure 8c,d also support this observation, showing that the thermal decomposition temperature of the PE-PDA-AlOOH increased from 409 °C to 451 °C relative to the PE separator. The DSC curves show that the PE separator begins to absorb heat at 125 °C, while the PE-PDA-AlOOH separator starts absorbing heat at 128 °C and the PE/AlOOH separator at 129 °C. Additionally, the heat absorption peak area of the PE separator is much larger than that of the PE/AlOOH and PE-PDA-AlOOH separators, indicating that the thermal stability of the PE-PDA-AlOOH and PE/AlOOH separators is similar to and significantly stronger than that of the PE separator. This improvement is attributed to the uniform grafting of AlOOH using covalent bonds. However, the slight difference in thermal stability between the PE-PDA-AlOOH separator and the PE/AlOOH separator is due to the incorporation of a portion of PDA and silane coupling agent.

**Figure 9:**
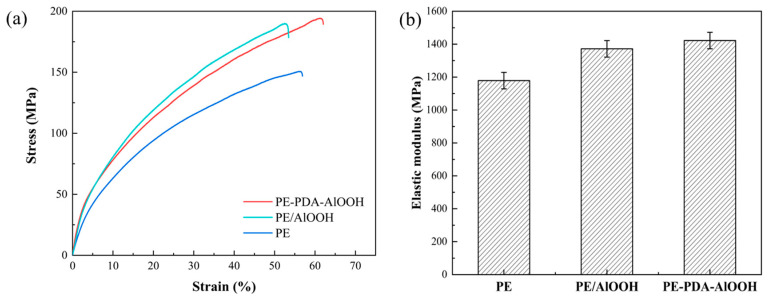
The stress/strain curves of different separators (**a**) and the elastic modulus (**b**).

The mechanical properties of the separator are also important indicators of battery safety. The stress–strain curves and elastic moduli of different separators were tested, as shown in Figure 9a,b. From the graph, it is evident that the fracture elongation of the PE-PDA-AlOOH membrane is 62.0% and the tensile strength is 194 MPa, which is significantly higher than that of the PE membrane (fracture elongation of 56.5% and tensile strength of 149 MPa) and the PE/AlOOH membrane. While the tensile strength of the PE/AlOOH membrane reaches 189 MPa, its fracture elongation decreases to 52%, indicating that although the rigidity of the PE/AlOOH membrane is improved, its flexibility and impact resistance are reduced compared to the PE membrane. As shown in Figure 9b, the elastic modulus of PE-PDA-AlOOH is the highest at 1422 MPa, indicating an improved ability to resist plastic deformation and enhanced stability of the separator. This is because the amino and phenolic hydroxyl groups in the covalent bonds formed in the PE-PDA-AlOOH membrane surface create stable chemical bonds, providing the membrane with some flexibility. This helps to disperse external stress concentration in the separator. 

**Figure 10:**
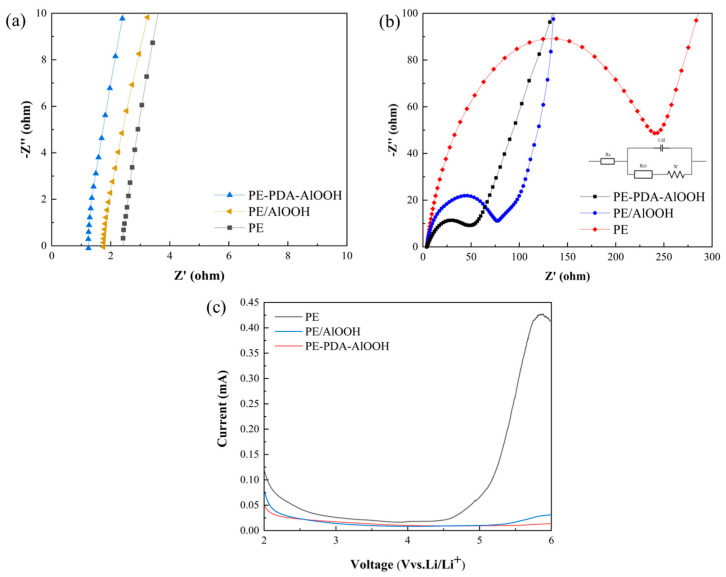
The bulk impedance (**a**), interface impedance (**b**), and LSV curves (**c**) of the different separators.

Ionic conductivity is one of the most crucial parameters for lithium-ion battery separators, as it is related to the internal resistance of Li^+^ passing through the separator. As shown in Figure 10a, the intercept on the Z’-axis in the curve represents the intrinsic impedance (R_b_) of the separator. Typically, the internal resistance of the separator is influenced by its thickness and microstructure. The addition of the γ-AlOOH layer increases the thickness, which, in turn, leads to an increase in the internal resistance of the separator [22]. In contrast, the increased microporosity provided by the γ-AlOOH layer and its improved wettability with the electrolyte can significantly reduce the internal resistance of the separator. The ionic conductivity of the separator can be calculated using Equation (3), as shown in Table 4. The ionic conductivity of PE-PDA-AlOOH is significantly higher than that of PE and PE/AlOOH. This is because the PE-PDA-AlOOH separator does not excessively increase the thickness of the membrane, and the covalent bonds formed contain a considerable number of hydroxyl and silane oxygen groups, which are hydrophilic functional groups capable of adsorbing more electrolyte, significantly increasing the Li^+^ content within the separator, thus enhancing the ionic conductivity of the separator. 

**Figure 11:**
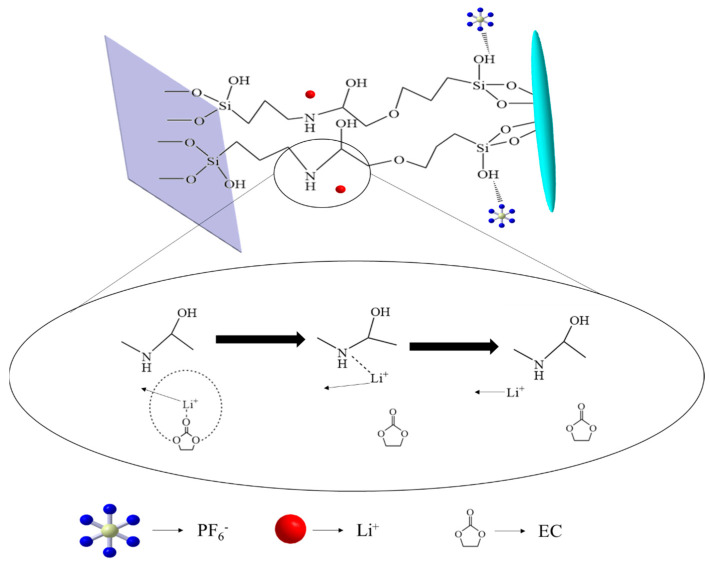
The process of the impact of covalent bonds on the migration of Li^+^.

In the electrolyte, the slightly negative oxygen atoms of carbonate esters (EC, DMC, and EMC) form a solvation shell around Li^+^ ions. This solvation shell expands the effective radius of lithium ions, thereby increasing the resistance to their movement within the electrolyte. Conversely, the lone pair of electrons on nitrogen atoms in covalent bonds can form temporary coordination bonds with the empty orbitals of Li^+^. When nitrogen atoms with their lone pairs form coordination bonds with lithium ions, they compete with the oxygen atoms of solvent molecules for lithium ions. This relatively weaker chemical bond can temporarily adsorb Li^+^ on the surface of the separator. Since the coordination bond is weaker compared to covalent bonds, Li^+^ can easily transition between adsorption and release, thus promoting a dynamic equilibrium of ions. This adsorption–release cycle facilitates the migration of Li^+^ within the separator, enhancing the ionic conductivity of the battery [49]. This process is illustrated in Figure 11.

Electrochemical impedance spectroscopy (EIS) tests were conducted on LiFePO_4_/Li batteries using different separators, and the resulting AC impedance spectra are presented in Figure 10b. The equivalent circuit diagram is shown in the inset of Figure 10b, with the specific values of the series resistance (Rs) and the interface impedance (Rct) for the PE, PE/AlOOH, and PE-PDA-AlOOH separators provided in Table 4. It can be observed that the Rs of the PE separator is 4.68 Ω, whereas for the PE-PDA-AlOOH and PE/AlOOH separators, it is 4.17 and 3.95 Ω, respectively. The interface impedance of the PE separator is 238.2 Ω. In contrast, the interface impedances of the PE-PDA-AlOOH and PE/AlOOH separators are only 51.0 Ω and 76.2 Ω, respectively, representing significant reductions. This phenomenon can be attributed to the fact that the electrolyte serves as the carrier of lithium ions, and the more electrolyte that is absorbed into the micropores of the separator and the greater the porosity, the lower the interface impedance of the separator. Moreover, the polar functional groups in the covalent bonds enable the separator to have better wettability with the electrolyte. Therefore, the PE-PDA-AlOOH separator exhibits the lowest interface impedance, consistent with the results obtained in Table 3.

According to the LSV curves in Figure 10c, the electrochemical stability windows for PE, PE/AlOOH, and PE-PDA-AlOOH were 4.57 V, 5.27 V, and 5.65 V, respectively. The PE-PDA-AlOOH separator exhibited the highest electrochemical stability window, indicating excellent electrochemical stability. This phenomenon can be ascribed to the presence of -OH and -NH groups in the separator, which absorb PF_5_ from the thermal decomposition of the electrolyte and HF generated from the reaction between the electrolyte and water. This absorption effectively inhibits electrolyte decomposition, ensuring the safety of battery applications. The PE-PDA-AlOOH separator with covalent grafting better facilitates the rapid migration of Li^+^. This is associated with the interaction between the hydrogen atom of the hydroxyl group in the covalent bond and the fluorine atom in the PF_6_^−^ anion of the LiPF_6_ electrolyte through an H-F bond, forming hydrogen bonds.

**Figure 12:**
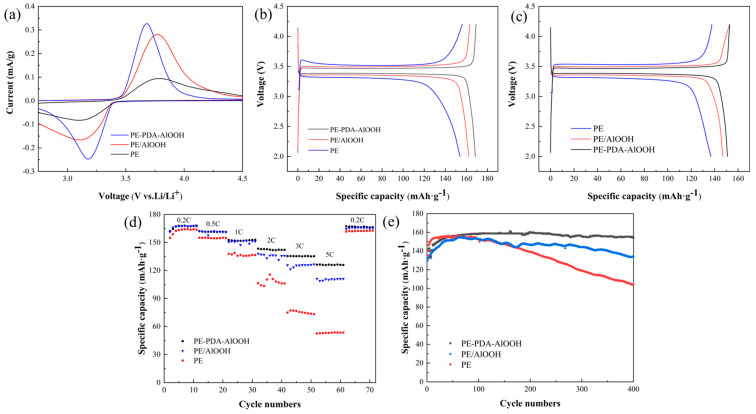
The CV curves (**a**), 0.1 C charge–discharge curves (**b**), 1 C charge–discharge curves (**c**), rate performance curves (**d**), and 2 C cycle for 400 cycles (**e**) of LiFePO_4_/Li half-cells assembled with different separators.

The cyclic voltammetry (CV) test of the LiFePO_4_/Li half-cell can analyze the reversibility and polarization of the battery system. The specific CV curves are shown in Figure 12a. It can be observed that each curve exhibits distinct oxidation–reduction peaks with good symmetry. A smaller potential difference at the same scanning rate implies a lower degree of irreversibility and polarization during the electrochemical reaction process [50]. The potential difference of PE-PDA-AlOOH is 0.57 V, significantly smaller than that of PE (0.68 V) and PE/AlOOH (0.65 V), indicating that the covalently grafted PE-PDA-AlOOH system exhibits better electrochemical reversibility and lower polarization. In addition, the peak current of the half-cell assembled with the PE-PDA-AlOOH membrane is much higher than that of the PE membrane, indicating that the modified separator has superior electrode conductivity compared to the PE separator. The absence of any peak impurities in the displayed CV curves suggests that the separator has excellent electrochemical inertness and does not participate in the battery’s redox reactions. These advantages ensure the normal operation of the battery, prevent side reactions, and contribute to improving the battery’s cycling and rate performance.

Figure 12b illustrates the comparative curves of the initial charge–discharge specific capacities under 0.1 C test conditions for the different separators. It is evident that the initial discharge specific capacity of the PE separator is 154 mAh/g, whereas that of the PE/AlOOH separator reaches 162.3 mAh/g, and the initial discharge specific capacity of PE-PDA-AlOOH reaches 168.6 mAh/g. This increase is attributed to the introduction of -OH in γ-AlOOH, which enhances the initial charge–discharge specific capacity of the battery. All the modified separators exhibit good porosity and liquid absorption rates, facilitating the rapid conduction of lithium ions between the positive and negative electrodes. Moreover, the PE-PDA-AlOOH separator contains not only -OH but also a large number of lithium-affinitive groups, such as pyrrole nitrogen, which promotes the formation of a stable SEI film between the separator and the electrode, thereby improving the battery’s cycling performance.

Figure 12c shows the comparative curves of the initial charge–discharge specific capacities under 1 C test conditions for different separators. It can be observed that the initial discharge specific capacity of the PE separator is 136.7 mAh/g, while the PE/AlOOH separator reaches 147 mAh/g, and the PE-PDA-AlOOH separator achieves 151 mAh/g. Compared to the 0.1 C test conditions, the initial charge–discharge specific capacities of all separators are reduced. This is due to the higher discharge rates requiring the battery to rapidly release the same amount of energy in a shorter period, which leads to greater heat generation and higher internal resistance inside the battery, thus affecting the overall energy output efficiency and capacity performance. However, the PE-PDA-AlOOH separator still shows the best discharge capacity performance.

Figure 12d shows the rate performance test results of different separator batteries at 0.2, 0.5, 1, 2, 3, and 5 C rates, followed by a return to a 0.2 C rate for 10 cycles. From the figure, it is evident that the average specific capacities for the PE separator at 0.2, 0.5, 1, 2, 3, and 5 C rates are 164, 154, 135, 108, 75, and 53 mAh/g, respectively; for PE/AlOOH, they are 167, 161, 147, 136, 125, and 110 mAh/g; and for PE-PDA-AlOOH, they are 168, 161, 151, 141, 135, and 126 mAh/g. The rate performance of the PE-PDA-AlOOH separator surpasses that of the PE and PE/AlOOH separators, especially at 5 C, where the capacity retention of PE-PDA-AlOOH is 75%, significantly higher than PE (32.6%) and PE/AlOOH (62.4%). Additionally, the figure shows that PE/AlOOH is unstable, with peeling of the AlOOH on the surface of the separator. In contrast, PE-PDA-AlOOH is more stable and reliable. This is attributed to the strong adhesion of the covalent bonds in the separator and the presence of a large number of polar functional groups, which improve the wetting and liquid absorption of the electrolyte by the separator, provide more channels for lithium-ion transport, effectively reduce interface impedance, decrease ion transport resistance, and thereby enhance the rate performance of the battery.

To determine the cycling performance and lifespan of the separator in the half-cell, a half-cell was assembled and cycled 400 times under 2 C testing conditions for analysis. The specific cycling performance is shown in Figure 12e. With an increase in the number of cycles, a significant decrease in the discharge specific capacity of the PE separator can be observed. As can be seen from the figure, the discharge specific capacity of the PE separator decreases from 156.4 mAh/g to 103.6 mAh/g, with a capacity retention rate of 66.2%; for the PE/AlOOH separator, the discharge specific capacity decreases from 154.7 mAh/g to 134 mAh/g, with a capacity retention rate of 86.6%; while for the PE-PDA-AlOOH separator, the discharge specific capacity decreases from 158.8 mAh/g to 154.2 mAh/g, with a capacity retention rate of 97.1%. As the number of cycles increases, the discharge specific capacity gradually decreases. Clearly, after 400 cycles, the PE-PDA-AlOOH separator exhibits a higher discharge specific capacity retention rate. This is because the modified separator has a higher ionic conductivity, better electrolyte wetting, a better liquid absorption rate, and superior interface stability when applied in lithium-ion batteries.

Table 5 presents the main materials, methods, and conclusions for typical AlOOH-modified PE separators in recent years. Overall, the covalently bonded graft-modified PE-PDA-AlOOH separator proposed in this article has advantages in terms of wettability, stability, and battery energy density, among other comprehensive performance aspects.

## 4. Conclusions

This paper proposes a novel approach to prepare a high-performance and stable PE-PDA-AlOOH separator by covalently bonding KH560-functionalized AlOOH onto PDA and KH550-modified PE membranes. This method overcomes issues such as inorganic particle detachment and the increase in coating thickness commonly observed in coated separators. As a lithium-ion battery separator, the covalent bonding enhances the interfacial stability of PE-PDA-AlOOH, while the -NH_2_ groups in the membrane facilitate electrolyte storage. This imparts good thermal stability, high electrolyte affinity, excellent mechanical properties, and high ion conductivity to the PE-PDA-AlOOH. When used as a separator in lithium-ion batteries, the LiFePO4/Li battery exhibits outstanding rate performance, with a discharge capacity of 126 mAh/g at a 5 C discharge rate. Furthermore, after 400 charge–discharge cycles, the capacity retention rate remains at 97.1%, with no observed detachment of AlOOH. Therefore, this covalently designed composite membrane structure provides valuable insights for overcoming the drawbacks of coated separators.

## Figures and Tables

**Table 1:** The elemental content in AlOOH-KH560.

Element	Al	O	Si	C
wt%	42.5	49.1	1.9	6.6

**Table 2:** The elemental content in PE-PDA-AlOOH.

Element	Al	O	Si	C
wt%	38.2	49.4	2.4	10.0

**Table 3:** Thickness, porosity, contact angle, and liquid absorption rate of the separators.

Parameter	Thickness (μm)	Porosity (%)	Contact Angle (°)	Liquid Absorption Rate (%)
PE	12 ± 1	41 ± 5	35 ± 2	107 ± 10
PE/AlOOH	23 ± 2	57 ± 4	11 ± 1	150 ± 12
PE-PDA-AlOOH	15 ± 2	67 ± 4	<5 ± 1	164 ± 10

**Table 4:** Impedance parameters and ionic conductivity of the different separators.

Parameter	Rb (Ω)	Ionic Conductivity (mS cm^−1^)	Rct (Ω)	Rs (Ω)
PE	2.4 ± 0.2	0.24 ± 0.03	238.2 ± 15	4.68 ± 0.7
PE/AlOOH	1.8 ± 0.3	0.58 ± 0.04	76.2 ± 5	4.17 ± 0.5
PE-PDA-AlOOH	1.2 ± 0.2	0.66 ± 0.05	51.0 ± 5	3.95 ± 0.5

**Table 5:** The main materials, practices, and conclusions regarding AlOOH-modified PE separators of recent years.

Main Materials and Methods	Thickness (μm)	Contact Angle (°)	Thermal Stability	Ionic Conductivity (mS cm^−1^)	Cycle Stability (%) (No. of Cycles, C Rate)	Refs.
AlOOH whiskers	25	8.6	No change at 150 °C	1.08 (PE: 0.85)	92% (100, 8 C)	[39]
AlOOH particles	18	Near 0	No change at 170 °C	0.65	94% (100, 1 C)	[28]
AlOOH with particle sizes	26	14.7		0.76 (PE: 0.27)	92% (100, 8 C)	[51]
1.4 μm AlOOH particles	26	5.7	No change at 170 °C	1.0 (PE: 0.55)	75.1% (200, 1 C)	[27]

## Data Availability

Data are contained within the article.
